# Supplementary material for: Comparing Performance of Spectral Image Analysis Approaches for Detection of Cellular Signals in Time-Lapse Hyperspectral Imaging Fluorescence Excitation-Scanning Microscopy
Source: Bioengineering (Basel). 2023 May 25;10(6):642. doi: 10.3390/bioengineering10060642 (PMC10295298; doi:10.3390/bioengineering10060642)
Supplement: Supplementary file 1 [file bioengineering-10-00642-s001.zip › Supplemental Figure S1.pdf]

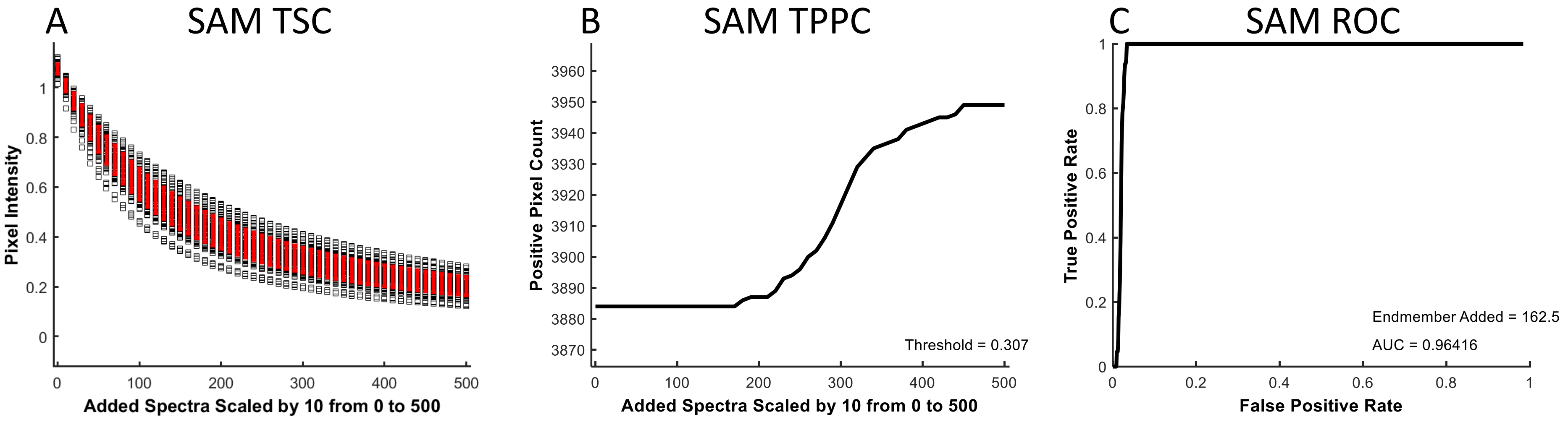

Supplemental Figure S1: Additional theoretical sensitivity analysis for an alternative range of values for the spectral angle mapper (SAM) algorithm. Three curves were calculated: the Theoretical Sensitivity Curve (TSC), Thresholded Positive Pixel Curve (TPPC), and Receiver Operator Characteristic (ROC) curve. When compared to Figure 6, D-F, a more stringent threshold for positive pixel detection results in decreased false positives (B), but also decreased ability to detect weak, true positive pixels. Reduced ability to detect weak true positive pixels is reflected in the Endmember Added value of 162.5 needed to generate a comparative area under the curve (AUC) for the ROC response (C).
